# Adoptive Transfer of Induced-Treg Cells Effectively Attenuates Murine Airway Allergic Inflammation

**DOI:** 10.1371/journal.pone.0040314

**Published:** 2012-07-09

**Authors:** Wei Xu, Qin Lan, Maogen Chen, Hui Chen, Ning Zhu, Xiaohui Zhou, Julie Wang, Huimin Fan, Chun-Song Yan, Jiu-Long Kuang, David Warburton, Dieudonnée Togbe, Bernhard Ryffel, Song-Guo Zheng, Wei Shi

**Affiliations:** 1 Developmental Biology and Regenerative Medicine Program, Department of Surgery, Children's Hospital Los Angeles, Los Angeles, California, United States of America; 2 Division of Rheumatology, Department of Medicine, The Keck School of Medicine, University of Southern California, Los Angeles, California, United States of America; 3 Institute of Immunology, Shanghai East Hospital of Tongji University, Shanghai, China; 4 Department of Respiratory Medicine, The Second Affiliated Hospital of Nanchang University, Nanchang, Jiangxi Province, China; 5 Molecular Immunology, University and CNRS, Orleans, France; Leiden University Medical Center, The Netherlands

## Abstract

Both nature and induced regulatory T (Treg) lymphocytes are potent regulators of autoimmune and allergic disorders. Defects in endogenous Treg cells have been reported in patients with allergic asthma, suggesting that disrupted Treg cell-mediated immunological regulation may play an important role in airway allergic inflammation. In order to determine whether adoptive transfer of induced Treg cells generated *in vitro* can be used as an effective therapeutic approach to suppress airway allergic inflammation, exogenously induced Treg cells were infused into ovalbumin-sensitized mice prior to or during intranasal ovalbumin challenge. The results showed that adoptive transfer of induced Treg cells prior to allergen challenge markedly reduced airway hyperresponsiveness, eosinophil recruitment, mucus hyper-production, airway remodeling, and IgE levels. This effect was associated with increase of Treg cells (CD4^+^FoxP3^+^) and decrease of dendritic cells in the draining lymph nodes, and with reduction of Th1, Th2, and Th17 cell response as compared to the controls. Moreover, adoptive transfer of induced Treg cells during allergen challenge also effectively attenuate airway inflammation and improve airway function, which are comparable to those by natural Treg cell infusion. Therefore, adoptive transfer of *in vitro* induced Treg cells may be a promising therapeutic approach to prevent and treat severe asthma.

## Introduction

Allergic airway inflammation and airway hyperresponsiveness (AHR) are characteristics of atopic asthma pathophysiology. More than 7% of Americans suffer from asthma [1], and annual expenditure for health and lost productivity due to asthma is estimated at nearly $20 billion. The currently available therapeutic approaches for asthma usually include quick symptomatic relief measures directed to relaxation of airway smooth muscle (bronchodilator) and long-term control with suppression of airway inflammation [2]. However, these existing standard asthma therapies have several caveats and remain inadequate. For example, inhaled anti-inflammatory corticosteroids only suppress but do not cure asthmatic inflammation, and long-term use of corticosteroids causes many pleiotropic side effects. Other more recently developed therapies, including inhibitors of leukotriene production and leukotriene receptor blockade, and anti-IgE monoclonal antibody (Omalizumab), are used as alternative treatments for persistent asthma. However, limited efficacy, high cost, and lack of responsiveness in some asthma patients are the major drawbacks. Thus, novel and more effective therapeutic approaches for asthma are still needed.

Recent studies have found that immune function dysregulation is one of the key pathogenic mechanisms underlying asthma [3]. Reduction and/or defects in regulatory T (Treg) cells, which function as negative regulators to suppress excessive immune response and maintain immunological tolerance have been detected in asthma patients [4]. Therefore, replenishment of Treg cells is thought to be a promising cell therapeutic approach. However, the use of thymus-derived naturally occurring regulatory T (nTreg) cells has several caveats that may significantly diminish their practical application for asthma treatment. These include limited availability, susceptibility to inflammation-triggered apoptosis, inability in suppressing pro-inflammatory Th17 cells, and self-conversion to Th17 and/or other T effector cells in the milieu of inflammation. In contrast, Treg cells that are induced by TGF-β and IL-2 in combination with low dose antigen exposure have similar phenotypic and functional characteristics to nTreg cells, without the caveats of nTreg cells mentioned above [5]. Herein, we report that adoptive transfer of the induced-Treg (iTreg) cells to OVA-sensitized mice either before or even after allergen challenge effectively attenuates OVA-induced airway allergic inflammation, AHR, and other asthma-like lung pathology by modulating the systemic immune system.

## Results

### Adoptive transfer of *in vitro* TGF-β-induced Treg (iTreg) cells prior to OVA challenge effectively prevented allergic inflammation in mouse respiratory airways and alveoli

**Figure 1:**
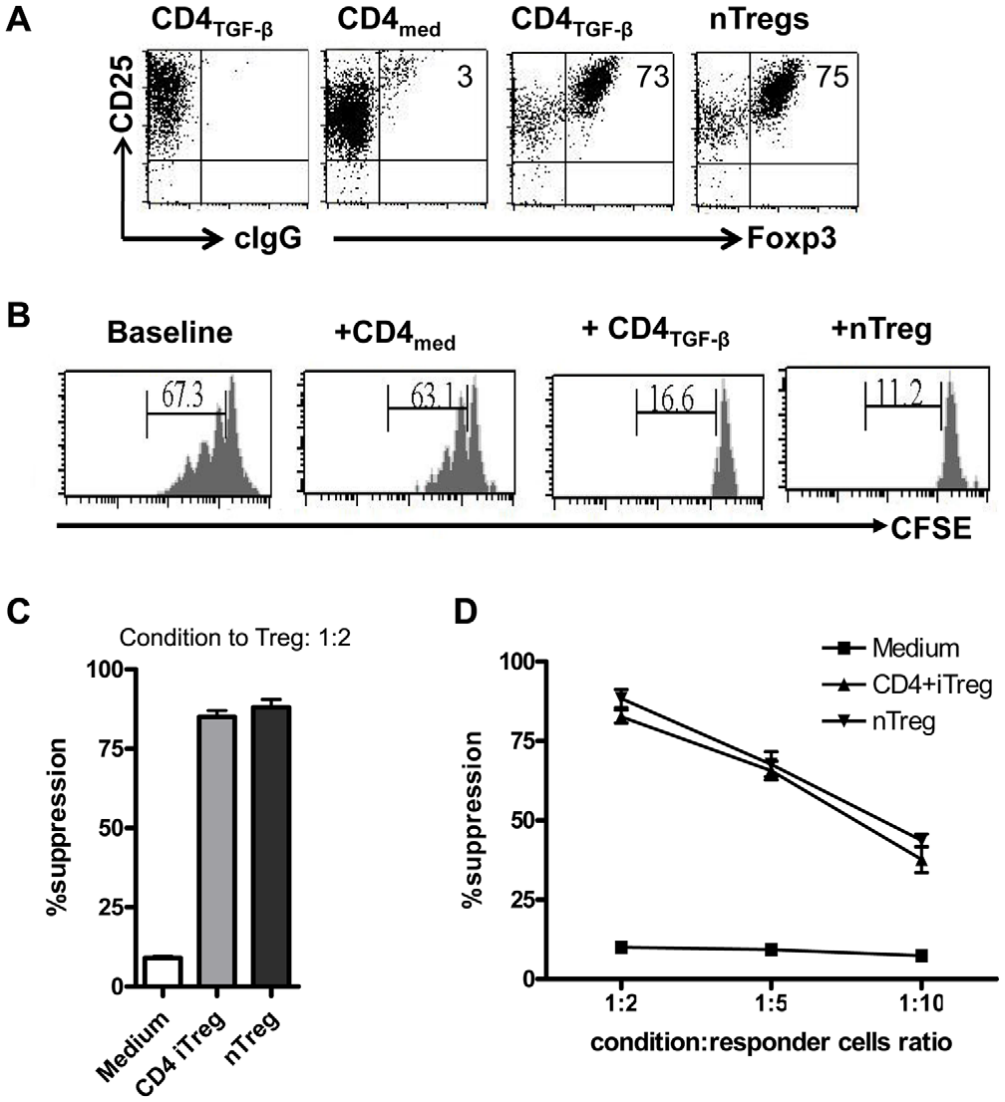
*In vitro* induction of regulatory T (iTreg) cells by TGF-β. Naive CD4^+^CD25^−^ cells were stimulated with anti-CD3/CD28 coated beads with IL-2 in the presence (CD4_TGF-β_) and absence (CD4_med_) of TGF-β for 5–6 days. nTreg cells were splenic CD4^+^CD25^+^ cells that were sorted and expanded with anti-CD3/CD28 coated beads with IL-2 for 6–7 days. (A). FoxP3 expression was determined by flow cytometry with anti-Foxp3 antibody. cIgG, control IgG. (B). T cells labeling with CFSE were stimulated with anti-CD3 with or without CD4 condition cells (ratio of CD4 condition to T responder = 1∶2) for three days and CFSE dilution was identified on the CD4^+^ cell gate. (C). T cell proliferation was determined by ^3^H-thymidine incorporation assay. (D). The T cell proliferation was determined in the different ratios of CD4 conditioned cells and T responder cells. Data was representative or mean ± SEM of three independent experiments.

iTreg cells were induced from splenic naïve CD4^+^CD25^−^ cells *in vitro* with TGF-β, IL-2 and anti-CD3/28 antibodies, as described previously [6]. As show in Fig. 1, more than 70% of the cells were induced to become iTreg cells. The phenotypes and functions of these iTreg cells are similar to those of nTreg cells (Fig 1).

**Figure 2:**
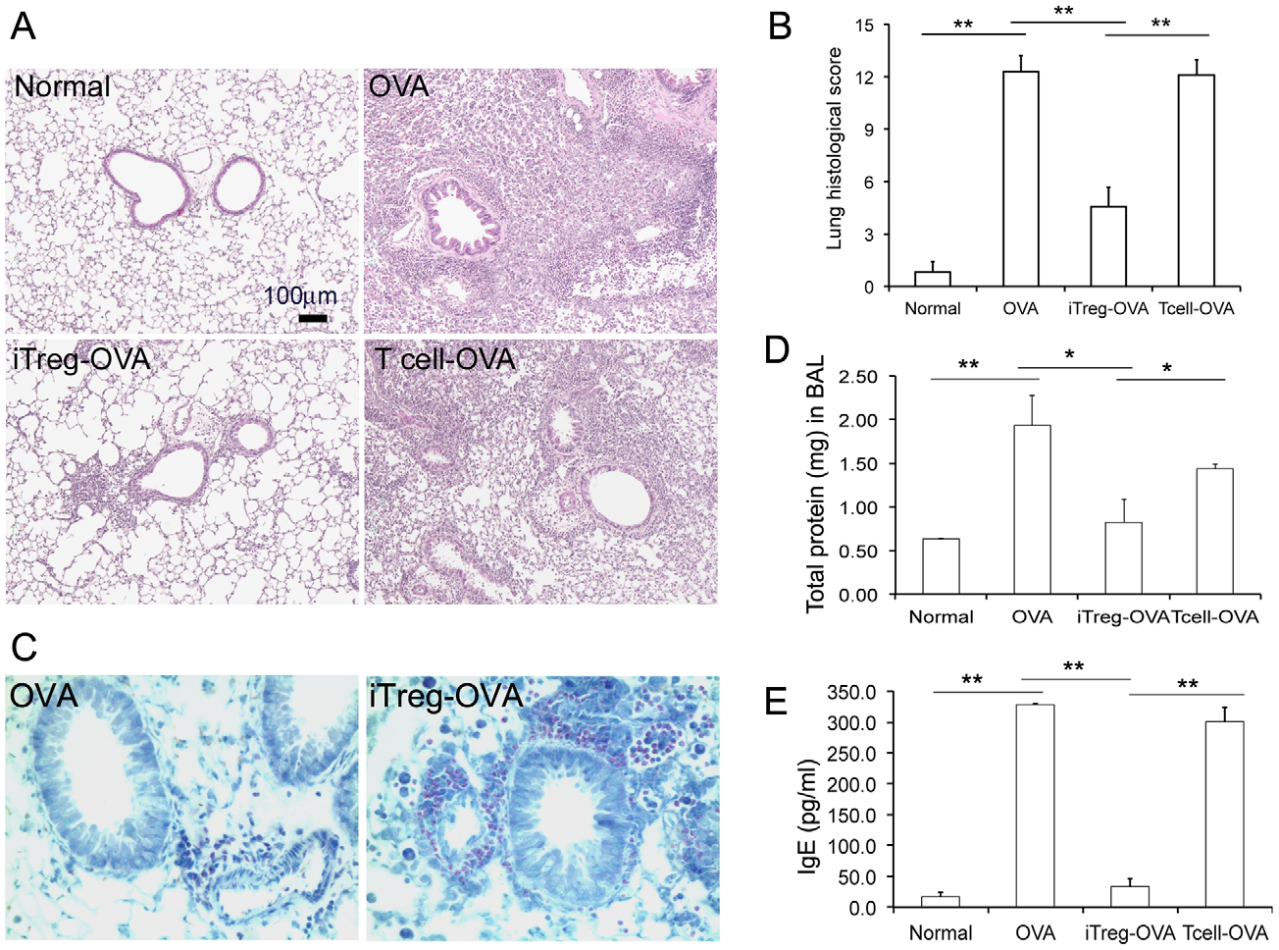
Attenuated allergic inflammation in lung tissues by adoptive transferring of iTreg cells prior to OVA challenge. (A) Lung tissue sections from the mice with indicated treatments were stained with H&E. (B) Overall lung inflammation were graded with scores 0 to 15 (none to severe inflammation, see Materials and Methods for details). (C) Eosinophil, detected by Discombe's staining (red intracellular granules), was the major type of cells that were infiltrated in small airways and adjacent vasculature. (D) Total proteins in BAL fluids from mice with different treatments were quantified. (E) IgE level in serum from different groups of mice was quantified by an ELISA. *P<0.05, **P<0.01, n = 5.

**Figure 3:**
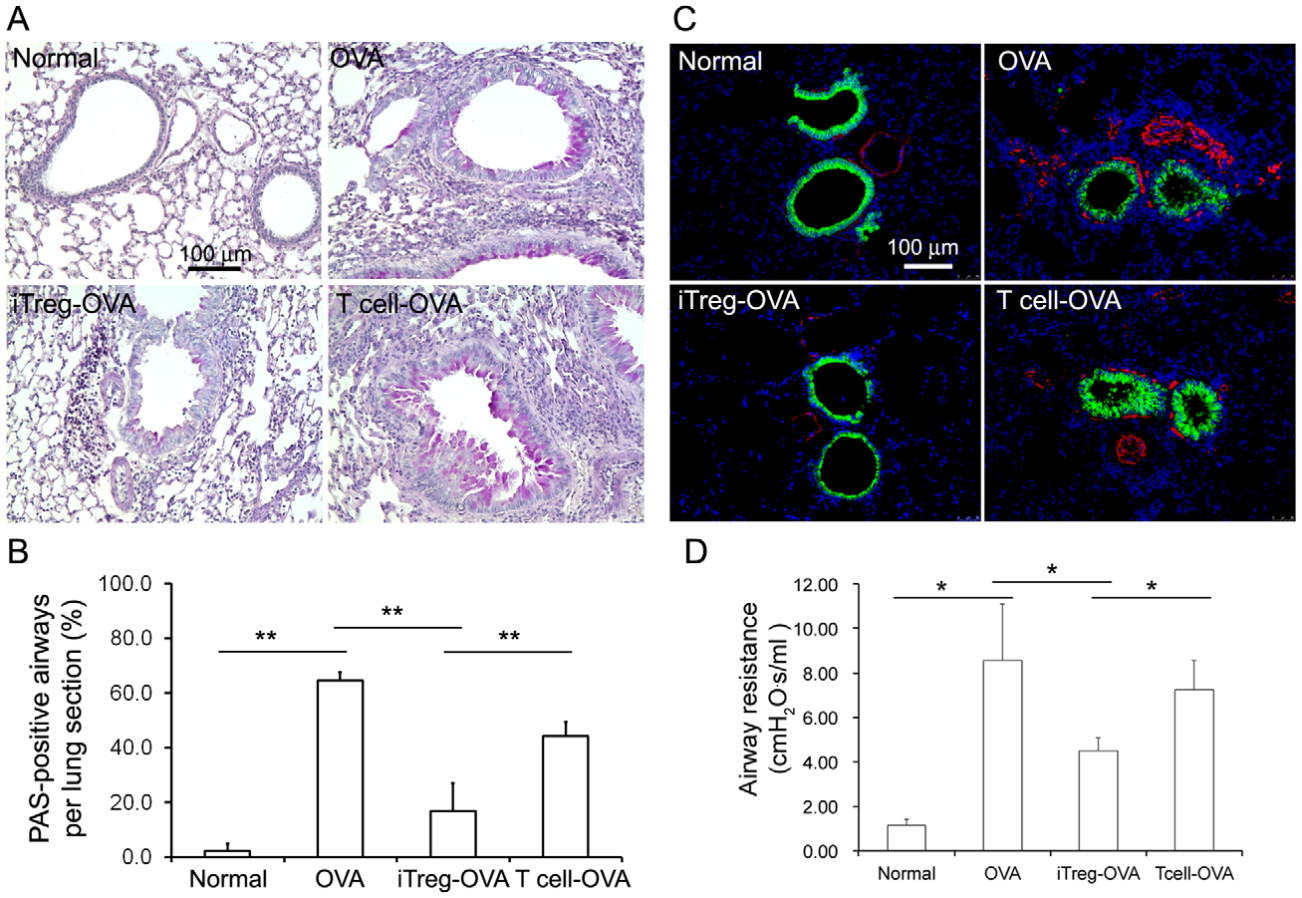
Abnormal airway wall remodeling and AHR were subsided with iTreg cell treatment. (A) Excessive mucin expression in small airway epithelial cells was detected by PAS staining (red color). (B) The numbers of airways with PAS-positive epithelial cells per lung tissue section were quantified in different experimental groups (n = 5). (C) Clara cells in small airway epithelia were stained by CCSP immunofluorescence staining (green) and the surrounding airway smooth muscle cells were immunostained by SMA (red). (D) Airway resistance was measured upon Mch (40mg/ml) aerosol delivery. Although the airway resistance was still significantly higher in iTreg cell-treated group than that in normal control group, significant reduction of airway resistance was achieved in iTreg cell-treated group compared to non-treated (OVA) or control T cell-treated group. *P<0.05, **P<0.01.

In OVA-sensitized mice, repeated intra-nasal (i.n.) ovalbumin (OVA) challenges at day 25–27 resulted in severe peri-vascular/peri-bronchiolar and alveolar inflammation, indicated by excessive inflammatory cell infiltration surrounding small airways and vasculature, as well as alveolar septa (Fig. 2A, 2B). The serum level of IgE was significantly increased, and infiltration of eosinophil around airway was also verified by Discombe's staining (Fig. 2C, 2E). Consistent with the lung histological changes, the total amount of proteins in bronchoalveolar lavage (BAL) fluid was significantly increased (Fig. 2D). The number of cells in BAL also increased more than 10-fold than the control groups (data not shown). Moreover, epithelial cell hypertrophy with increased mucin expression in small airways, thickened airway smooth muscle cell (SMC) layer, and resultant smaller lumen with rippled epithelial surface of small airways were also observed in OVA-challenged mouse lungs (Fig. 3). Therefore, a typical OVA-allergic airway inflammatory model was verified.

Using this established OVA-allergic mouse model, the preventive anti-inflammatory effect of adoptive transfer of iTreg cells was first examined. Three days before OVA challenge (Day 22), a single transfer of 5×10^6^ iTreg cells was given to the mice via tail vein injection. T cells cultured without TGF-β addition were used as an additional specificity control. After three-day i.n. OVA challenge, lung specimens were harvested for detailed analyses. iTreg cells, but not control T cells, significantly attenuated OVA-induced allergic inflammation including reduced infiltration of inflammatory cells, particularly eosinophils, in airway and alveolar septa, decreased levels of serum IgE and BAL proteins (Fig. 2), as well as reduction in the number of cells in BAL by 2-fold (p<0.05). Alterations of airway walls subsequent to allergic inflammation, including epithelial hypertrophy and increased mucin production (PAS-positive staining), as well as thickened smooth muscle cells in small airways (Fig. 3A, 3B, 3C), were likewise significantly attenuated. These results indicate that adoptive transfer of iTreg cells prior to OVA allergic challenge can effectively prevent lung inflammation and abnormal airway remodeling.

### Adoptive transfer of iTreg cells prior to OVA challenge effectively inhibited airway hyperresponsiveness (AHR) in OVA-challenged mice

OVA-sensitized mice with repetitive i.n. administration of OVA developed significant AHR to methacholine (MCh) challenge compared to normal control mice (Fig. 3D). However, adoptive transfer of iTreg cells prior to repetitive challenge of OVA significantly inhibited AHR, although increased AHR was still detected. In contrast, adoptive transfer of T control cells did not significantly reduce AHR, although slight reduction in AHR was detected in some mice. Thus, adoptive transfer of iTreg cells, but not control T cells, prior to allergen exposure can also effectively reduce AHR in addition to the reduction in airway inflammation described above.

### Adoptive transfer of iTreg cells prior to OVA challenge modulated immune response to OVA-allergen

**Figure 4:**
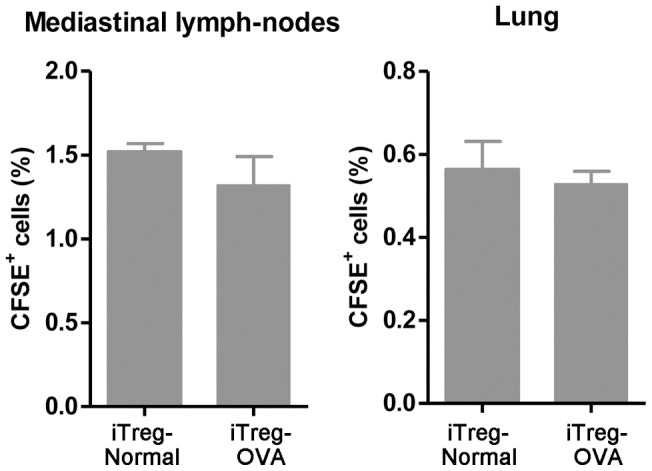
Comparison of infused CFSE-labeled iTreg cells in the absence and presence of OVA-induced allergic inflammation. No significant difference was detected in mediastic draining lymph nodes and lung tissues for infused iTreg cells between normal control mice and mice that had OVA-induced allergic inflammation.

The infused exogenous iTreg cells, labeled with CFSE (carboxyfluorescein succinimidyl ester) dye, were detected 8 days after injection in mediastinal draining lymph nodes and lung tissues with comparable frequencies between normal and OVA-challenged mice (Fig. 4), suggesting that adoptively transferred iTreg cells are homing to these tissues independent of pulmonary allergic inflammation.

**Figure 5:**
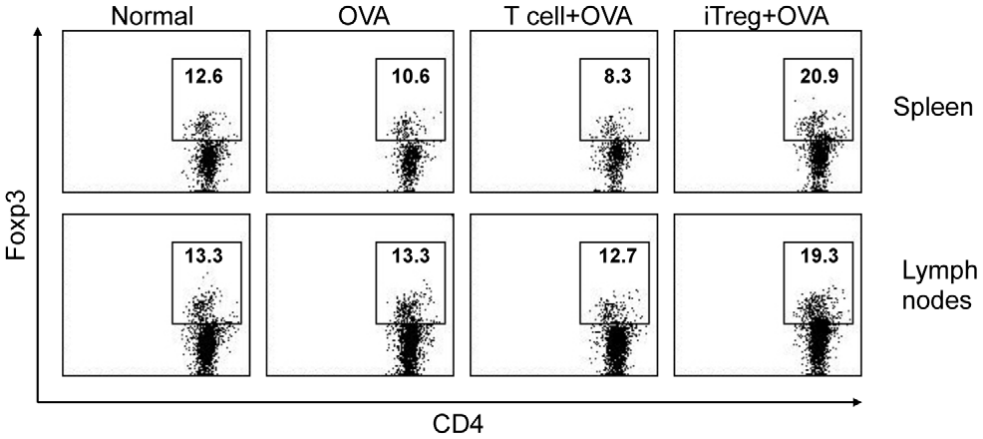
Increased total CD4^+^FoxP3^+^ Treg cells in the spleen and draining lymph nodes of the mice that received exogeneous iTreg cells. In contrast, the frequencies of CD4^+^FoxP3^+^ Treg cells did not show significant changes between normal mice and OVA mice, as well as control T cell-treated mice. The experiments were repeated with consistent results.

**Figure 6:**
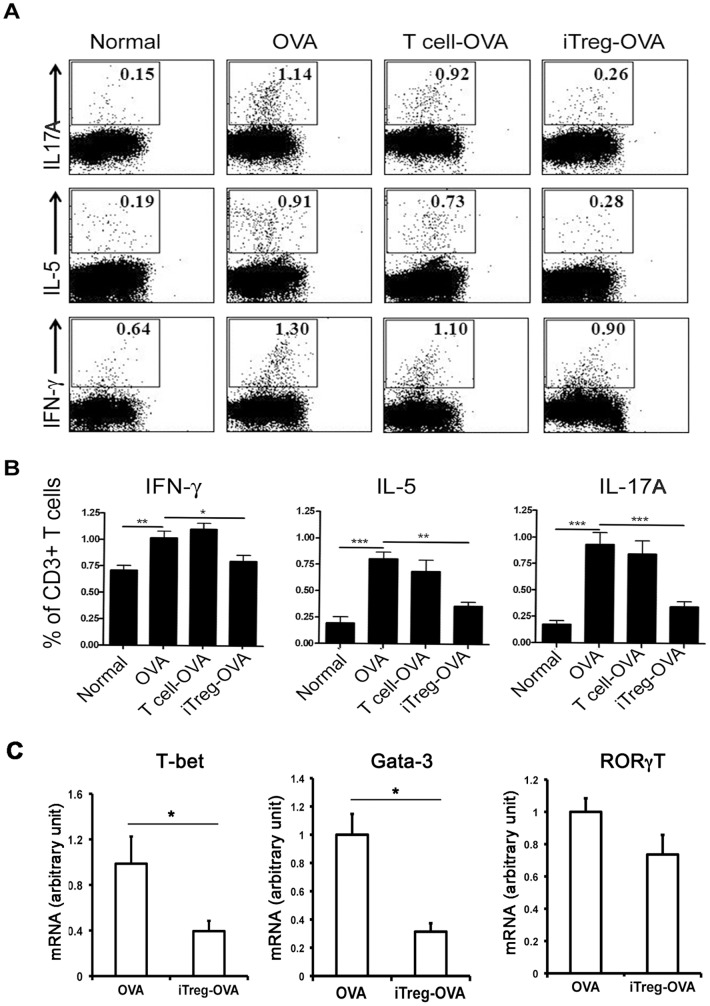
Adoptive transfer of iTreg cells significantly diminished Th1/Th2/Th17 cell frequencies in draining lymph nodes in asthmatic mice. Intracellular expression of IFN-γ, IL-5, and IL-17A in CD3^+^ T cells were determined by FACS. (A) A representative of 9 mice in each group. Cells were gated on CD3^+^ cells. (B) Results were mean ± SEM of values of 9 mice in each group. *P<0.05, **P<0.01, ***P<0.001. (C) Significant reductions of T cell differentiation markers, including T-bet1 and Gata-3, but not RORγT, were detected in iTreg-treated OVA mice compared to iTreg-untreated OVA control.

Numerous studies have found that asthmatic inflammation is related to abnormal cellular immunity, including defective Treg cells, inappropriate ratio of Th2 to Th1 cell population, and dysfunction of Th17 and dendritic cells (DCs). Thus, we have compared these immune cells and their related cytokine production between iTreg cell-treated and untreated OVA-asthmatic mice. In OVA-asthmatic mice that received exogenous iTreg cells prior to OVA challenge, about 2-fold increase of Treg cells (CD4^+^FoxP3^+^) was detected in the draining lymph nodes and the spleen, while there were no significant changes in the number of Treg cells in untreated or control T cell-treated OVA group (Fig. 5). In mediastinal draining lymph nodes, increased Th1, Th2, and Th17 cells caused by repetitive OVA challenge was significantly attenuated by adoptive transfer of iTreg cells (Fig. 6A, 6B), but not by control T cells. Moreover, in OVA-challenged mouse lung tissues, increases expression of Th1 and Th2 differentiation-related transcription factors T-bet1 and Gata-3 was also significantly inhibited by infused iTreg cells, as measured at the mRNA level (Fig. 6C). However, slight but not statistically significant reduction of RORγT, a transcription factor related to Th17 differentiation, was detected in iTreg-treated OVA mouse lung compared to untreated OVA control. This suggests that adoptive transfer of iTreg cells may suppress Th1 and Th2 cell differentiation in OVA mouse lungs.

**Figure 7:**
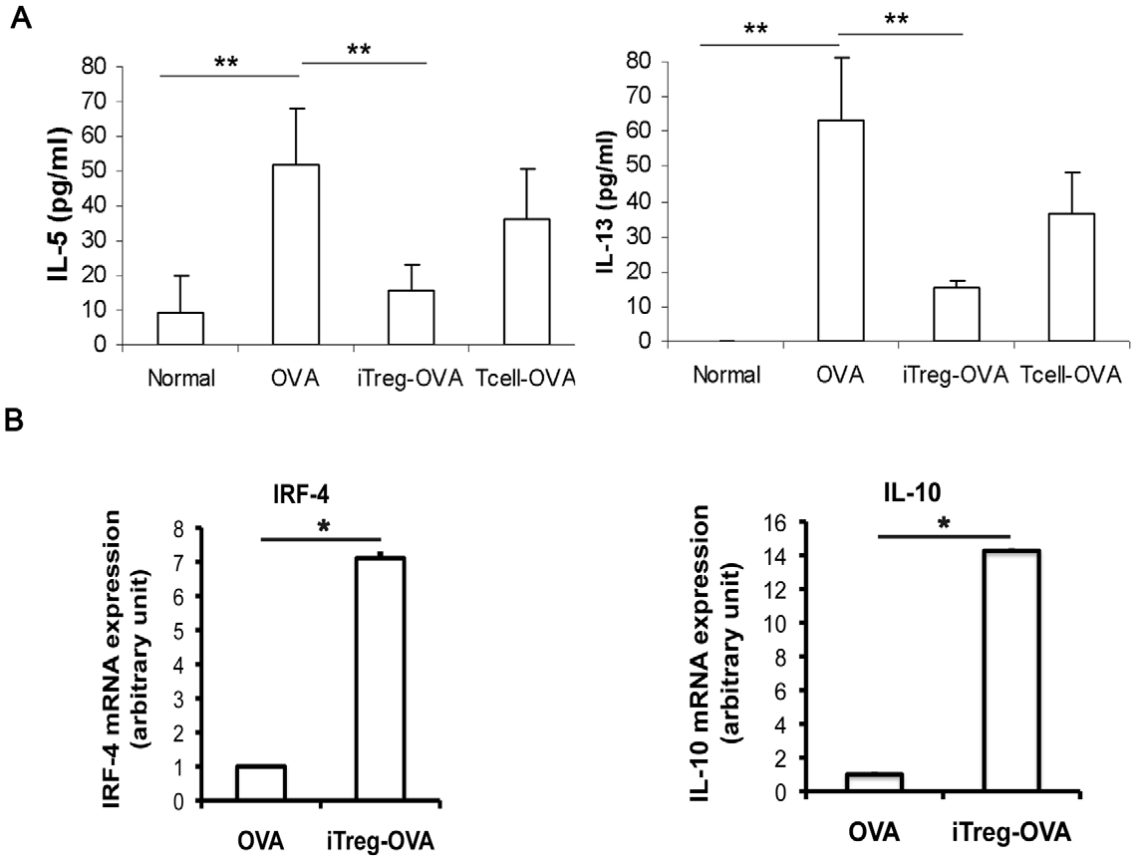
Adoptive transfer of iTreg cells altered cytokine production. (A) iTreg cells inhibited OVA-induced increase of Th2 cytokines. IL-5 and IL-13 in mouse serum were quantified by specific ELISA. Significant reduction of IL-5 and IL-13 in the group receiving iTreg treatment was detected compared to OVA challenge only control group. **P<0.01. (B) Expression of IRF-4 and IL-10 at the mRNA level of lung lymphocytes was significantly increased in iTreg-treated OVA mice compared to untreated OVA mice. *P<0.05.

Consistent with the above changes, serum Th2 cytokines including IL-5 and IL-13 were significantly increased upon OVA challenge (Fig. 7A). Adoptive transfer of iTreg cells, but not T control cells, was able to partially block these increases in mice. Recently studies reported that a crucial role of Treg cells in suppressing Th2-driven mucosal inflammation, and the inhibitory properties of the Treg cells were mediated by enhancing expression of IRF-4 and IL-10 [7]–[9]. Interestingly, a 7-fold increase of IRF-4 mRNA and a 14-fold increase of IL-10 mRNA expression were also detected in total lymphocytes isolated from iTreg cell-treated OVA lung compared to those from untreated OVA mice (Fig. 7B).

**Figure 8:**
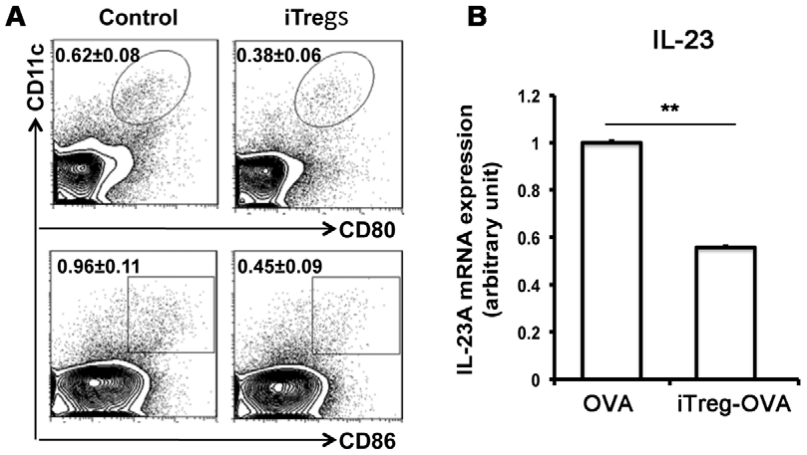
Alteration of dentritic cells and related cytokine IL-23. Significant reduction of both CD11c^+^CD86^+^ and CD11c^+^CD80^+^ subsets of DCs in mediastinal lymph nodes were detected in iTreg-treated OVA mice compared to untreated OVA mice. (B) IL-23 expression at the mRNA level in draining lymph nodes was also significantly reduced by iTreg treatment compared to untreated OVA mice (Fig. 8B).

Since migration of respiratory tract DCs to the draining mediastinal lymph node was reported to be an important mechanism to activate antigen-specific T cells during airway allergic inflammation [10], the effect of adoptively transferred iTreg cells on alteration of DCs in the OVA-challenged mice were investigated. Compared to untreated OVA-challenged mice, expression of both CD80^+^ and CD86^+^ of CD11c^+^ cells in mediastinal lymph nodes were significantly attenuated by infused iTreg cells (Fig. 8A). Moreover, the level of DC-related cytokine IL-23 expression in draining lymph nodes was also significantly reduced by injected iTreg cells compared to untreated OVA mice (Fig. 8B). This suggests that suppression of DC-initiated allergic response may be one of the mechanisms underlying iTreg-mediated attenuation of airway allergic inflammation. Therefore, adoptive transfer of iTreg cells may modulate both local and systemic immunity, and thus prevent excessive cellular response against OVA-induced allergic reaction and inflammation.

### Adoptive transfer of iTreg cells during OVA challenge also effectively attenuated airway inflammation and reduced airway hyperresponsiveness

**Figure 9:**
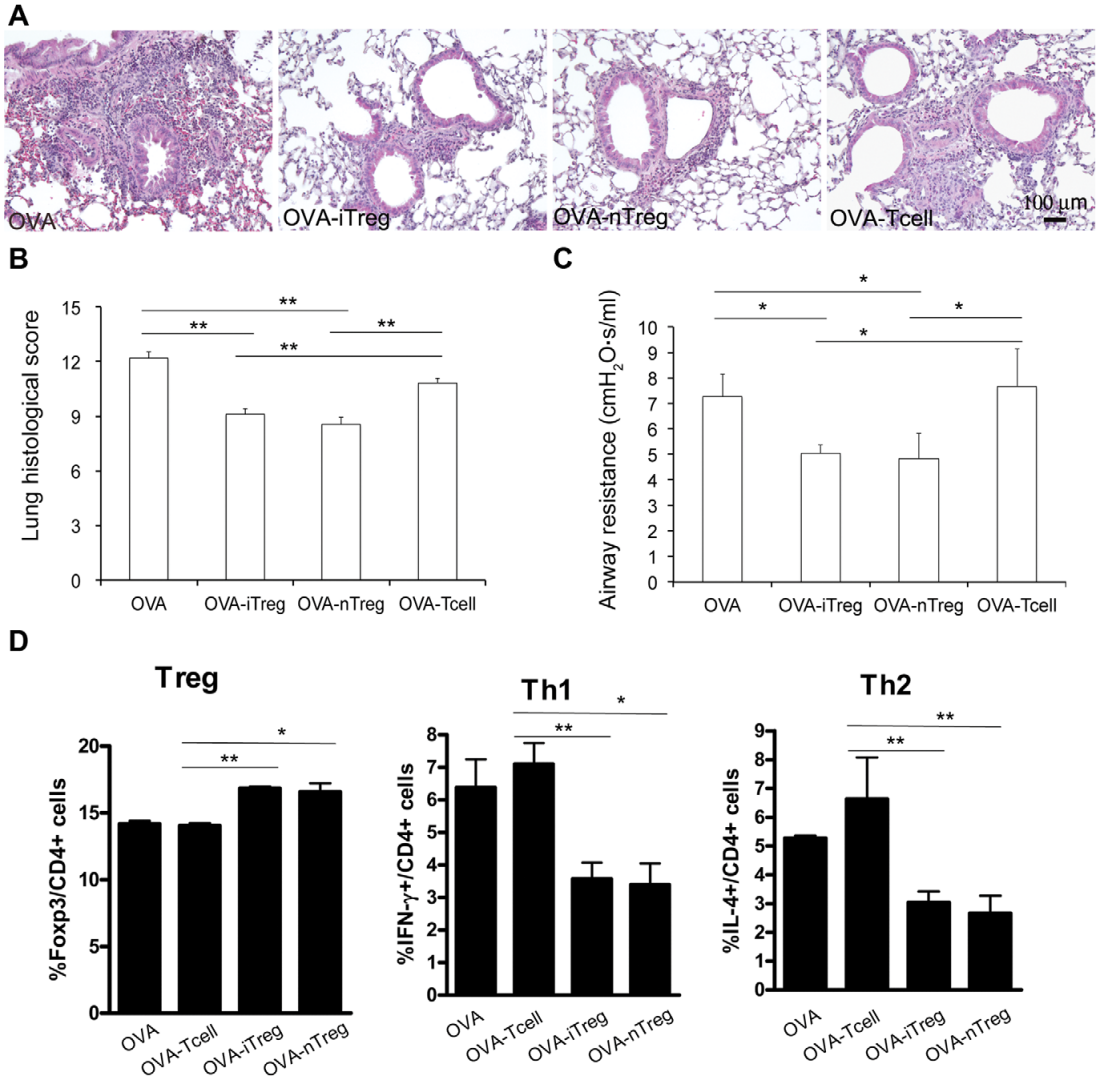
Adoptive transfer of Treg cells after first OVA challenge still effectively suppressed airway inflammation and AHR, as well as Th1/Th2 cell frequencies. (A) Histopathological changes of lungs from the mice that received no cell, iTreg, nTreg, or control T cells after first OVA challenge (day 25). Lung specimens were taken on day 28 after another two daily OVA challenges on day 26 and 27. (B) Accumulated inflammatory scores of peri-vascular, peri-bronchiolar and alveolar regions of different groups. (C) Airway resistance was measured to evaluate lung functional changes among different groups. (D) Altered frequencies of Treg, Th1, and Th2 subsets in the spleens of the mice receiving different treatments. *P<0.05, **P<0.01.

In addition to the effects of iTreg cells in preventing allergen-induced airway inflammation and ARH, we also evaluated the therapeutic potential of adoptive transfer of iTreg cells by giving iTreg cells after first allergic challenge. Interestingly, the lung inflammation, particularly in peri-vascular and peri-bronchiolar area, was significantly attenuated in the mice received iTreg cells injection compared to those of the OVA-challenged mice received no cells or control T cells (Fig 9A). Moreover, adoptive transfer of expanded nTreg cells also had reduced lung inflammation, comparable to that in iTreg-treated lung. The inflammatory histopathology was further quantified using the same scale described above (Fig 9B). Both iTreg and nTreg cell treatments had comparable anti-inflammatory effects in this acute allergic inflammatory model. Consistent with the changes in lung inflammation, AHR to Mch was also significantly attenuated in the mice that received either iTreg or nTreg injection after first time OVA challenge (Fig 9C). In addition, both iTreg and nTreg cell treatment significantly increased Foxp3+ Treg cells and decreased Th1 and Th2 cell frequency in spleens (Fig. 9D), draining lymph nodes and lungs (data not shown). Conversely, treatment with CD4 control cells did not cause significant changes in Treg and Th1 cell frequency, while it increased Th2 cell numbers in the OVA-asthma mouse model (Fig. 9D). As IL-17A and IL-17F cell frequency was very low in the OVA-asthma mice (data not shown), we were not able to detect alteration of Th17 cell frequency after cell therapy. This suggests that iTreg cells generated *in vitro* have therapeutic effects comparable to nTreg cells in controlling asthma progression. Thus, the use of both Treg cell subsets could provide an effective approach to diminish the allergic inflammation already occurred in the lung, and to improve airway functions.

## Discussion

Asthma is an inflammatory disorder of the conducting airways with abnormal airway hyper-responsiveness and remodeling, resulting in airflow restriction during breathing [3], [11]. Studies have found that immune function dysregulation is one of the key pathogenic mechanisms underlying airway allergic inflammation, in particular imbalance between Th2 cell and Th1 cell responses [12], [13]. More recently, another pivotal subset of CD4^+^ T cells (Treg cells), which have been related to control immune tolerance and subsequent autoimmune diseases, were also found to be important in suppressing allergic responses [14], [15]. For example, CD4^+^CD25^+^ Treg cells from grass pollen-allergic individuals were less able to suppress proliferative responses and IL-5 production by CD4^+^CD25^−^ T cells [16]. Significant reduction of the CD4^+^CD25^+^ Treg cell frequency in peripheral blood was detected in patients with persistent or exacerbation of asthma when compared to control groups [17]. In addition, Treg cells in patients with asthma were also decreased in bronchoalveolar lavage (BAL) fluid [18], [19]. In mouse asthma models, naturally occurring Treg (nTreg) cells are present in the lung tissue of sensitized mice and increase upon allergen inhalation. Inhibition of nTreg cells augments respiratory allergen-induced AHR and IgE production, as well as Th2 cytokine levels in BAL fluid [20]. Therefore, cell therapy by replenishing functional Treg cells may be a new promising strategy for asthma prevention and treatment. It also have advantages by correcting the immune cell proportions and functions, which restore appropriate cytokine productions, compared to just targeting a single or a few cytokines produced by these dysregulated immune cells.

Treg cells can be either derived from the thymus (nTreg cells), or induced in the periphery in a TGF-β-dependent fashion (iTreg cells) [4], [21]. Both nTreg and iTreg cells share similar phenotypic characteristics and immune suppressive functions. nTreg cells are rare cell population so that it is difficult to obtain sufficient numbers of nTreg directly for the therapeutic need. Although repetitive expansion *in vitro* is able to generate enough nTreg cells, recent study has indicated that the phenotypes and functional characteristics of the nTreg cells after the repetitive expansion have been altered [22]. Moreover, intrinsic CD4^+^CD25^+^ nTreg cells are often defective in suppressing allergic immune responses in asthma patients, which limits the clinical use of these nTreg cells as autograft therapeutic agents. Our study has shown that iTreg cells are effective in both preventing and treating airway allergic responses, and that *in vitro* induced-iTreg has a comparable anti-inflammatory activity to that of nTreg cells. iTreg cells may migrate to inflammatory sites in airways, and likely suppress Th1 and Th2 immune response directly or indirectly through inhibiting DC cells.

Induction of iTreg cells *in vitro* not only avoids systemic application of cytokines and growth factors, but also generates an approach similar to an autograft, by which sufficient iTreg cells can be easily induced from the CD4^+^ cells in individual asthmatic patient *in vitro*, and then adoptively transferred back to the same patient to induce immune tolerance to allergens and to suppress abnormal inflammatory responses in the airway without significant side effects. Moreover, iTreg cells also have several superior functional features compared to nTreg cells, including anti-apoptosis and resistance to Th17 conversion [21], [23]. Conversely, nTreg cells are more plastic and unstable under pro-inflammatory conditions [23], [24]. Therefore, adoptive transfer of iTreg cells may be a better approach as a novel cell therapy in airway allergic diseases.

Although many studies have demonstrated that adoptive transfer of iTregs can control lupus, colitis, gastritis and diabetes in animal models [25]–[29], it is unknown whether infusion of iTreg cells can attenuate airway allergic inflammation and improve respiratory function after asthma onset, We have now shown that adoptive transfer of iTreg cells before allergen challenge effectively prevented airway allergic inflammation and improved airway function in an OVA model. In addition to local relief of inflammation, abnormal systemic immune functions were also corrected to some extent. Of importance, the infusion of iTreg cells during allergen challenge still had significant effects on reducing lung inflammation and ARH, although to a less degree compared to iTreg cell administration prior to allergen challenge. The different efficacies between these two regimens can be caused with these possibilities. (1) Exogenous iTreg cells are more effective to attenuate the initiation of the inflammatory process, but less effective for the inflammation already occurred; (2) It needs certain time for infused iTreg cells to be effective, as all lung specimens were taken on day 28, regardless of when iTreg cells were given on day 22 before allergen challenge or on day 25 after first allergen challenge. Nevertheless, both iTreg and nTreg cells displayed the similar therapeutic effects on asthma progression. Mechanistically, Treg subset infusion regulates the local and systemic immune balance by increasing Treg and decreasing Th1/Th2 cell frequencies in the ongoing asthma.

In summary, adoptive transfer of *in vitro* induced iTreg cells is an effective way to both prevention and treatment of airway allergic disease, such as asthma, in a mouse OVA-asthma model, which will be a promising therapeutic approach for airway allergic diseases.

## Methods

### OVA-sensitized mouse asthma model and exogenous cell infusion

6 to 8-week-old female C57BL/6 mice weighing 20–25g were used for the experiments. Mice were sensitized by intraperitoneal (i.p.) injections of 25 µg OVA mixed with aluminum hydroxide (Pierce) at day 1, and followed by another booster i.p. injection at day 14. These sensitized mice then were challenged with 20 μg of OVA through an intranasal (i.n.) route for three consecutive days (days 25, 26, and 27). 5×10^6^ of Treg cells or control T cells were intravenously injected into mice before allergen challenge (day 22), or after first allergen challenge (Day 25). Lung functional test and specimen were all performed 24 hours after last allergen challenge (day 28). Experiments were approved by IACUC at Children's Hospital Los Angeles.

### *Generation of in vitro* TGF-β-induced regulatory T (iTreg) cells

Splenic CD4^+^CD25^−^D62L^+^CD44^low^ naive T cells were isolated by autoMACS (Miltenyi Biotech) from the littermates to the mice used for generating the OVA-asthma model. iTreg cells were then prepared as previously described [6]. Briefly, CD4^+^CD25^−^ cells were treated with anti-CD3/CD28 coated beads and IL-2 in the presence of TGF-β for 5 days, the TGF-β-induced Treg cells (CD4^+^CD25^+^ FoxP3^+^) cells were then sorted by flow cytometry.

#### Isolation and expansion of natural Treg (nTreg) cells

Splenic CD4^+^CD25^+^ cells were sorted and expanded with anti-CD3 and CD28 coated beads as described previously [30]. Foxp3 expression of nTregs was more than 75% and sustained the suppressive activity after expansion (Fig. 1).

### Determination of airway hyperresponsiveness (AHR) *in vivo*

The mice were anesthetized with i.p. injection of sodium pentobarbital (90mg/kg). A tracheostomy was performed. The mice were then connected to a computer controlled small animal ventilator (FlexiVent, SCIREQ) and ventilated at 150 breath/min with a tidal volume of 10ml/Kg and a positive end-expiratory pressure of 3 cmH_2_O. Methacholine (MCh, 40 mg/ml in PBS) was then delivered to the subject by nebulized aerosol. The frequency-independent airway resistance in mouse lung was measured by FlexiVent/SCIREQ software, and the MCh challenge experiments are repeated at least three times.

### Lung histopathology and immunohistochemistry

Discombe's and Periodic-Acid-Schiff staining were used detect eosinophil and glycoprotein, respectively [31]. Lung inflammation was evaluated using a semi-quantitative method [32]. Inflammation foci were scored 0–5 (0 = no foci, 1 = ≤5, 2 = 6–15, 3 = 16–25, 4 = 26–35, 5 = ≥35) separately for peri-vascular and peri-bronchiolar, as well as alveolar regions. Thus, the total inflammation score is the sum of these three measurements in a range of 0–15. At least four mice in each group were selected for this analysis, as presented by mean ± SEM.

### Serum and intracellular cytokine analysis

The levels of cytokines (IL-4, IL5 and IL-13) and IgE in sera were measured using ELISA kits (Invitrogen). Lymphocytes in axillary draining lymph nodes and spleen were collected and stained for markers. In the case of intracellular IL-4, IL-17A and IFN-γ, cells were _stimulated_ with 0.25 μg/ml PMA and 0.25 μg/ml ionomycin (Calbiochem), and followed by incubation with brefeldin A (5 μg/ml) for additional 4 hours. The phenotypes and intracellular cytokine expression were analyzed using a LSRII Flow Cytometry.

### Real-Time PCR analysis and primers

Total tissue RNAs were isolated form snap-frozen lung tissue or lymph nodes using a RNeasy kit (Qiagen, Valencia, CA). Synthesis of cDNA and quantitative reverse transcriptase analysis were performed using iScript cDNA synthesis kit, and real-time quantitative PCR was performed using SYBR Green I and iCycle-iQ system (Bio-Rad) as previously published [33]. The PCR primers were: T-bet (5′-TCAACCAGCACCAGACAGAG-3′, 5′-AAACATCCTGTAATTGGCTTGTG-3′), Gata3 (5′-CTTATCAAGCCCAAGCGAAG-3′, 5′-CCCATTAGCGTTCCTCCTC-3′), RORγT (5′-CACTGCCAGCTGTGTGCT-3′, 5′-TGCAAGGGATCACTTCAATTT-3′), IRF-4 (5′-CAATGTCCTGTGACGTTTGG-3′, 5′-GTTCCTGTCACCTGGCAAC-3′), IL-10 (5′-ACTGCACCCACTTCCCAGT-3′, 5′-TGTCCAGCTGGTCCTTTGTT-3′), IL-23A (5′-CACCAGCGGGACATATGAA-3′, 5′-CCTTGTGGGTCACAACCAT-3′), respectively. GAPDH was used to normalize equal addition of template cDNAs.

### Statistical analysis

The quantitative data are presented by mean ± SEM. Statistical analyses were performed by ANOVA. Differences were considered statistically significant when a P value is less than 0.05.
